# Generation of Bacterial Diversity by Segregation of DNA Strands

**DOI:** 10.3389/fmicb.2021.550856

**Published:** 2021-03-22

**Authors:** Vic Norris, Camille Ripoll

**Affiliations:** ^1^Laboratory of Microbiology Signals and Microenvironment, Faculty of Science, University of Rouen, Mont Saint Aignan, France; ^2^Faculty of Science, University of Rouen, Mont Saint Aignan, France

**Keywords:** heterogeneity, bacteria, chromosome segregation, cell cycle, model, hyperstructure, assembly

## Abstract

The generation in a bacterial population of a diversity that is coherent with present and future environments is a fundamental problem. Here, we use modeling to investigate growth rate diversity. We show that the combination of (1) association of extended assemblies of macromolecules with the DNA strands and (2) the segregation of DNA strands during cell division allows cells to generate different patterns of growth rate diversity with little effect on the overall growth rate of the population and thereby constitutes an example of “order for free” on which evolution can act.

## Introduction

Bacterial cells live in unpredictable environments in which they must be able to both profit from opportunities and survive dangers. An individual bacterium cannot simultaneously be ready to grow quickly in favorable conditions and to grow slowly (or not grow at all) in unfavorable ones. The solution that some species have adopted is for genetically identical bacteria in the same population in the same environment to grow with different rates; this is despite the population itself growing in steady state in that environment for generations. Such growth rate diversity is a subset of the phenotypic diversity that characterizes many—if not all—species. Hence, understanding how growth rate diversity is generated may help explain how most phenotypic diversity is generated.

Phenotypic diversity is often attributed to stochasticity or noise at the level, for example, of the transcription of key genes during the life of the cell (Elowitz et al., 2002; Chen et al., 2020). However, noise alone may not suffice to generate reproducibly (1) at the level of the individual cell, a large set of constituents that act together in a phenotype that is coherent (i.e., appropriate, complementary and non-contradictory, as when there is generation of the degradosome, which contains both exo-ribonucleases and endo-ribonucleases) and (2) at the level of the population, a range of phenotypes that is coherent with the possible environments that may arise. An alternative or complementary hypothesis is that the cell cycle, which includes DNA replication, DNA segregation and cell division, is itself responsible for generating an environmentally appropriate diversity of coherent phenotypes (Norris and Amar, 2012).

The basis of the above cell cycle hypothesis for diversity generation is that bacteria are highly structured with much of their mass in the form of spatially extended assemblies of macromolecules that serve one more functions, alias *hyperstructures*, that have different characteristics (Norris et al., 2007). Types of hyperstructures include (1) those made from enzymes such as EF-Tu (Mayer, 2006; Defeu Soufo et al., 2010), CTP synthase (Ingerson-Mahar et al., 2010) and RNases (Taghbalout and Rothfield, 2007), (2) those involved in ribosome synthesis (Woldringh and Nanninga, 1985; Jin et al., 2017) and in DNA replication and segregation (Sunako et al., 2001; Boeneman et al., 2009; Duderstadt et al., 2010; Sanchez-Romero et al., 2011; Helgesen et al., 2015; Taniguchi et al., 2019), (3) those formed by the coupling of transcription, translation, and either insertion into membrane or into cytoplasmic complexes (Binenbaum et al., 1999; Llopis et al., 2010), (4) those generated by phase separation (Hondele et al., 2019) such as the clusters of RNA polymerase and NusA (Ladouceur et al., 2020) and (5) those involving the Nucleoid-Associated Proteins such as H-NS (Wang et al., 2011; Kuwada et al., 2015; Gao et al., 2017) and HU (Guo and Adhya, 2007; Qian et al., 2017; Datta et al., 2019; Weng et al., 2019).

Some of these hyperstructures are physically associated with the chromosome. For example, several studies are consistent with the existence of hyperstructures containing the rRNA operons, six out of seven of which are colocated (Gaal et al., 2016), along with nascent rRNA, RNA polymerase and NusA (Weng et al., 2019; Ladouceur et al., 2020). Moreover, the distribution of these hyperstructures is believed to be influenced by that of the nucleoid (Weng et al., 2019). It is worth noting here that certain mRNAs are located next to the genes that encode them (Llopis et al., 2010) and that, in eukaryotes, the same nucleus can contain both an active and an inactive nucleolus (Slodzian et al., 1992). In the strand segregation hypothesis, we proposed that, following DNA replication, each parental strand plus hyperstructures physically associated with it confers a particular phenotype on the daughter cell into which it is segregated (Rocha et al., 2003; Konto-Ghiorghi and Norris, 2020). Since these hyperstructures could be of the equilibrium or the non-equilibrium type, one daughter could receive a parental DNA strand plus equilibrium hyperstructures that steer it toward a slow growth phenotype whilst the other daughter could receive the other parental DNA strand plus non-equilibrium hyperstructures that steer it toward a fast growth phenotype (Norris et al., 2007). The distribution of genes on the DNA strands of two leading model bacteria, *Escherichia coli* and *Bacillus subtilis*, is consistent with this hypothesis (Rocha et al., 2003). In a recent investigation of growth rate diversity in bacteria using stable isotope-labeling and *Secondary Ion Mass Spectrometry*, *SIMS*, we have obtained additional evidence to support and extend the strand segregation hypothesis (Gangwe Nana et al., 2018). Here, we use modeling to determine whether the combination of DNA strand segregation and hyperstructure segregation operating within the cell cycle could indeed generate the diversity of growth rates that we and others observe (Calabrese et al., 2019) and hence whether this combination could underpin the generation of other forms of phenotypic diversity.

## Materials and Methods

### Growth of the Single Cell

**FIGURE 1:**
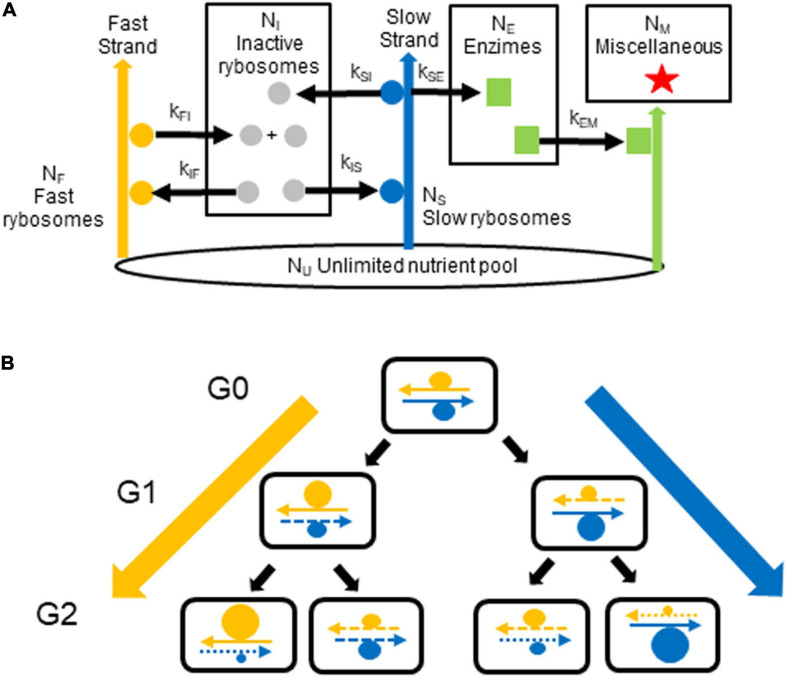
Generation of growth rate diversity. **(A)** The model. In each cell, fast rybosomes (orange circles) use the fast strand (orange arrow) to synthesize inactive rybosomes (gray circles) from nutrients. Some of these rybosomes are transferred, via the pool of inactive rybosomes, to the slow strand (blue arrow) to synthesize enzimes from nutrients; these rybosomes, therefore become the slow rybosomes (blue circles). Reciprocally, slow rybosomes that are transferred, via this pool of inactive rybosomes, to the fast strand become fast rybosomes. Enzimes (green squares) catalyze the production (green arrows) of miscellaneous material (red stars). The numbers of inactive rybosomes, fast rybosomes, slow rybosomes, enzimes, and miscellaneous material in the cell are N_I_, N_F_, N_S_, N_E_, and N_M_, respectively. The pool of nutrients, N_U_, is constant (black ellipse). The kinetic constants for the catalyzes (black arrows) are: k_FI_ and k_IF_ for the production of rybosomes; k_SI_, k_IS_, and k_SE_ for the production of enzimes (where k_SI_ equals k_SE_). **(B)** The hypothesis. In the first generation, G0, a fast hyperstructure (orange circle) and a slow hyperstructure (blue circle) are associated with the fast and slow strands, respectively. As these hyperstructures are segregated with these parental strands (continuous arrows) over the next generations, G1 and G2, they continue to increase in size in the cell lines on the extreme left and right (thick orange and blue arrows). Newly replicated strands are represented by dashed arrows where the length of the dashes indicates the generation in which the strand was synthesized.

In the model shown in Figure 1, one DNA strand encodes the cell’s transcriptional and translational resources (i.e., encodes RNA polymerase, ribosomes, tRNA synthetases, etc.) that are needed for fast growth; we term this strand the *fast* strand. The other strand encodes the enzymes needed for survival (e.g., for diverse metabolic pathways, structural material and reserves); we term this strand the *slow* strand. We use a single neologism, *rybosomes*, to designate all the different elements (transcription factors, RNA polymerase, etc.) which perform transcription so as to make RNA from DNA **plus** all the different elements (ribosomes, elongation factors, tRNAs, etc.) which perform translation so as to make proteins from some of this RNA (the messenger RNA). We term *fast rybosomes*, F, those rybosomes responsible for making the rybosomes themselves, which they do by using the genes on the fast strand. We term *slow rybosomes*, S, those rybosomes responsible for making the other constituents of the cell (such as the enzymes that catalyze general metabolism and the production of reserves), which they do by using the genes on the slow strand. We then term *inactive rybosomes*, I, those rybosomes that are not interacting with the strands. We term *enzimes*, E, all the proteins that catalyze metabolic reactions, that transport ions, that perform all the cellular functions (other than transcription and translation performed by rybosomes) and that in our model diffuse freely. Finally, we term *miscellaneous*, M, all the other constituents of the cell—that is, DNA, lipids, peptidoglycan, small molecules, ions, etc. These groups of “cellular constituents” in our model correspond roughly to the major groups of real constituents in *E. coli* (Bremer and Dennis, 2008).

Cellular constituents are synthesized from an unlimited pool of nutrients (e.g., nucleotides and amino acids), Nu, as shown in Figure 1. Note that, in our model, all rybosomes must be either “fast” because they are physically associated with a “fast DNA strand” or “slow” because they are physically associated with a “slow DNA strand” or “inactive” because they diffuse without association with the DNA strands. The synthesis of rybosomes, enzimes and miscellaneous material is given by:

(1) I→F

(2) N⁢u+F→2⁢I

(3) I→S

(4) N⁢u+S→E+I

(5) N⁢u+E→E+M

Equations (1) and (3) are not chemical transformations but represent the conversion of an inactive rybosome into a fast or a slow rybosome, respectively. Taken together, Eqs. (1) and (2) represent the autocatalytic synthesis of fast rybosomes. Equation (4) represents the synthesis of enzimes as catalyzed by slow rybosomes; after its synthesis, the enzime and slow rybosome are released and this rybosome becomes an inactive rybosome. Equation (5) represents the synthesis of miscellaneous material as catalyzed by enzimes. For simplicity, the reactions in Eqs. (2), (4), and (5) (which reflect a combination of many processes) are taken to go in only one direction, which corresponds to the absence of degradation in our model.

The only difference between the fast rybosomes and the slow rybosomes is whether they use the fast DNA strand or the slow DNA strand to make either the rybosomes or the enzimes, respectively. Both fast rybosomes and slow rybosomes must pass through a diffusible state as inactive rybosomes from which they can then become either fast or slow rybosomes.

Again, for simplicity, we consider the cell to be a system with five distinct phases (constituted by the F, S, I, E, and M species); hence the growth of the cell corresponds to the sum of the growth of the five phases, which in turn corresponds to the sum of the numbers of the five constituents N_SUM_ = (N_F_ + N_S_ + N_I_ + N_E_ + N_M_), where N_F_, N_S_, N_I_, N_E_, and N_M_ are the numbers of fast rybosomes, slow rybosomes, inactive rybosomes, enzimes and miscellaneous, respectively.

Using the formalism of chemical kinetics, the following differential equations, based on (1) to (5) represent the evolution of the system:

(6) $${\text{dN}}_{\text{I}}/\text{dt}=-\left(k_{1}+k_{3}\right).N_{\text{I}}+2\,k_{2}^{\prime }.N_{\mathrm{Nu}}.N_{F}+k_{4}^{\prime }.N_{S}$$

(7) $${\mathrm{dN}}_{F}/\mathrm{dt}=k_{1}.N_{I}-k_{2}^{\prime }.N_{\mathrm{Nu}}.N_{F}$$

(8) $${\mathrm{dN}}_{S}/\mathrm{dt}=k_{3}.N_{I}-k_{4}^{\prime }.N_{S}$$

(9) $${\mathrm{dN}}_{E}/\mathrm{dt}=k_{4}^{\prime }.N_{\mathrm{Nu}}.N_{S}$$

(10) $${\mathrm{dN}}_{M}/\mathrm{dt}=k_{5}^{\prime }.N_{\mathrm{Nu}}.N_{E}$$

where k_1_ and k_3_ are the kinetic constants of the first order, unidirectional, transformations (1) and (3), k′_2_, k′_4_, and k′_5_ the kinetic constants of the second order, unidirectional, transformations (2), (4), and (5). Assuming a constant pool of nutrients, N_*Nu*_, the transformations (2), (4), and (5) become degenerate first order transformations with kinetic constants:

$$k_{\mathrm{FI}}=k_{2}^{\prime }.N_{\mathrm{Nu}},k_{\mathrm{SI}}=k_{4}^{\prime }.N_{\mathrm{Nu}},k_{\mathrm{EM}}=k_{5}^{\prime }.N_{\mathrm{Nu}}$$

and for coherent notation:

$$k_{\mathrm{IF}}=k_{1},k_{\mathrm{IS}}=k_{3}$$

We can therefore rewrite equations (6) to (10) as:

(11) $${\mathrm{dN}}_{I}/\mathrm{dt}=-\left(k_{\mathrm{IF}}+k_{\mathrm{IS}}\right).N_{I}+2.k_{\mathrm{FI}}.N_{F}+k_{\mathrm{SI}}.N_{S}$$

(12) $${\mathrm{dN}}_{F}/\mathrm{dt}=k_{\mathrm{IF}}.N_{I}-k_{\mathrm{FI}}.N_{F}$$

(13) $${\mathrm{dN}}_{S}/\mathrm{dt}=k_{\mathrm{IS}}.N_{I}-k_{\mathrm{SI}}.N_{S}$$

(14) $${\mathrm{dN}}_{E}/\mathrm{dt}=k_{\mathrm{SI}}.N_{S}$$

(15) $${\mathrm{dN}}_{M}/\mathrm{dt}=k_{\mathrm{EM}}.N_{E}$$

Even though the system of Eqs. (11) to (15) is linear and can therefore be solved formally, we prefer for programming convenience solving it numerically using a simple iterative method:

$$N_{I}\left(\mathrm{iter}+1\right)=[-\left(k_{\mathrm{IF}}+k_{\mathrm{IS}}\right).N_{I}\left(\mathrm{iter}\right)+2.k_{\mathrm{FI}}.N_{F}\left(\mathrm{iter}\right)$$

(16) $$+k_{\mathrm{SI}}.N_{S}\left(\mathrm{iter}\right)]+N{}_{I}(\mathrm{iter})$$

$$N_{F}\left(\mathrm{iter}+1\right)=\left[k_{\mathrm{IF}}.N_{I}\left(\mathrm{iter}\right)-k_{\mathrm{FI}}.N_{F}\left(\mathrm{iter}\right)\right]$$

(17) $$+N_{F}\,\left(\mathrm{iter}\right)$$

$$N_{S}\left(\mathrm{iter}+1\right)=\left[k_{\mathrm{IS}}.N_{I}\left(\mathrm{iter}\right)-k_{\mathrm{SI}}.N_{S}\left(\mathrm{iter}\right)\right]$$

(18) $$+N_{S}\,\left(\mathrm{iter}\right)$$

(19) $$N_{E}\left(\mathrm{iter}+1\right)=\left[k_{\mathrm{SI}}.N_{S}\left(\mathrm{iter}\right)\right]+N_{E}\left(\mathrm{iter}\right)$$

(20) $$N_{M}\left(\mathrm{iter}+1\right)=\left[k_{\mathrm{EM}}.N_{E}\left(\mathrm{iter}\right)\right]+N_{M}\left(\mathrm{iter}\right)$$

Where iter = 0, 1, 2, 3… corresponds to the number of the iteration. The expressions in square brackets represent the mass increase of each species after one iteration. Initially, we choose a system in which: (1) an arbitrary value is given to each of the kinetic constants and the initial number of species, (2) the number of iterations, iter_MDT_, required for the sum of the species to double, is then given by N_SUM_(iter_MDT_) = 2. N_SUM_(0) using iteration equations (16–20), (3) the cell then divides and the five species, N_I_(iter_MDT_), N_F_(iter_MDT_), N_S_(iter_MDT_), N_E_(iter_MDT_), and N_M_(iter_MDT_) are shared equally between the two daughter cells, (4) a test is then applied: if N_I_(iter_MDT_)/2, N_F_(iter_MDT_)/2,… are significantly different from N_I_(0), N_F_(0),… then N_I_(0), N_F_(0),… are put equal to N_I_(iter_MDT_)/2, N_F_(iter_MDT_)/2,… and a new iteration is performed, (5) this process is repeated until near-equality for each species is obtained, which corresponds to an invariant point in terms of the numbers of the species in the newborn cell, (6) if this near-equality cannot be obtained, the values of the kinetic constants are all reduced by the same factor and the whole process is repeated.

To calibrate the system and to facilitate comparison of bacteria, the number of iterations, iter_MDT_, required for a cell to double in mass is multiplied by a calibration factor to give the MDT in minutes. This factor was obtained from experimental results for the composition of an *E. coli* cell growing in steady state with an MDT of around 64 min (the “medium” growth rate conditions, see Composition of the Average Cell); the calibration factor then equals 64/iter_MDT_ where the iter_MDT_ is for a cell in our system with a similar composition to a real cell. The same value of this calibration factor is then used to obtain the MDTs of cells grown with different initial compositions and/or with different values of the kinetic constants.

In biological reality there is a limit to the number of RNA polymerases that can bind to and transcribe a gene and to the number of ribosomes that can bind to and translate an mRNA; this means that the rate of synthesis of proteins is limited. Therefore, in our model, the synthesis of both rybosomes and enzimes is limited by limiting the maximum numbers of fast and slow rybosomes in the cell (see section “Implementation of the Model”). The division of the mother cell gives two daughter cells, each with mass N(0), which we set arbitrarily to 4,000 mass units. The composition of these daughter cells is determined by sharing out the fast, slow and inactive rybosomes, enzimes, and miscellaneous material present in the mother cell at the time of division.

### Segregation of Hyperstructures and Cell Division

When the mother cell divides, one daughter inherits a greater proportion of the fast rybosomes (and the other daughter a correspondingly greater proportion of the slow rybosomes) as determined by association of the rybosomal hyperstructures with the parental strands and by the semi-conservative nature of the principal mechanism of DNA replication and segregation; P(seg), the proportion of a hyperstructure segregating with a strand varies can take values between and including 0 and 1. Once these rybosomes have been segregated into the daughters, the enzimes are distributed with a probability that depends on the ratio of the spaces available in the daughters. This space depends on taking into account the minimum and maximum requirements for all the constituents. Finally, the remaining constituents are distributed into the daughters according to the space available. This segregation pattern continues over the generations.

### Growth of the Population in a Turbidostat

Firstly, a population of cells of random composition in terms of rybosomes, enzimes, and miscellaneous material is considered. Each cell is identified by a number. Each cell then grows and its mass doubling time (MDT) is a function of its composition. After a time has elapsed equal to its MDT, the cell has doubled in mass and becomes a mother cell that then divides. The contents of this mother cell are then distributed into its daughter cells to give two cells that may be different. In a turbidostat, the growth medium flows in and cells flow out (along with medium) such that the number of cells in the turbidostat remains constant; the growth of these cells is limited only by the capacity of the cells’ metabolism to use these nutrients (i.e., is not limited by the availability of nutrients). To simulate life in a turbidostat, when a mother cell divides, one of the daughter cells is identified by assigning to it the number of the mother cell whilst the other daughter cell is identified by assigning to it the number of a cell that is chosen at random to be replaced (which corresponds to the replaced cell flowing out of the turbidostat). To characterize the growth rate diversity of the population we use the average MDT, standard deviation and the coefficient of variation or CV (the standard deviation of the distribution divided by the arithmetic mean) because these are so widely used in biology though other measures of such diversity have recently been proposed (Calabrese et al., 2019). We calculate the distribution of the MDTs of the cells and the CV irrespective of the size of the cells. We also calculate the MDT of the population as the mean of the MDTs of the mass units of the individual cells, which entails multiplying the number of mass units in a cell by the MDT of that cell (thereby taking into account the size of each cell) and dividing by the number of mass units in the entire population. After a number of generations that depends on the initial conditions, the values of the MDTs and the CV become constant and the population is considered to be in steady state.

### Composition of the Average Cell

It is important to note that the range in the compositions of the average cell in different populations of bacteria is considerable: even for a single species, such as *E. coli*, the proportion of the cellular mass in the form of the transcriptional-translational machinery can vary dramatically; as Mass Doubling Times, MDTs, go from 20 to 100 min, the number of ribosomes per cell falls from 7.10^4^ to 8.10^3^ (Bremer and Dennis, 2008) whilst the proportion of these ribosomes that are active falls from 90% in fast growth to under 20% in slow growth (Dai et al., 2016). Moreover, *E. coli* can grow with the different rates in the same medium – and with the same rate but with different compositions in different media (Bremer and Dennis, 2008).

To calibrate our system, we focus on the well-characterized growth of *E. coli* in steady state in minimal medium plus glucose at 37°C where no factor is limiting; in these conditions the proportion of inactive ribosomes is around 10% of all ribosomes (Dai et al., 2016; Li et al., 2018), the MDT of the population, at least for the K-12 strain, is around 64 min and growth rate diversity is extensive (Gangwe Nana et al., 2018). For 10^9^ cells of the B/r strain of *E. coli* growing with an MDT of 60 min, the dry mass is **374** μg, of which total protein is 214 μg, RNA is 44 μg and DNA is 9.5 μg (Bremer and Dennis, 2008). Of this RNA, 98% is stable RNA, a major constituent of the translation machinery. Ribosomal proteins constitute 9.2% of total protein whilst the other proteins essential for transcription and translation constitute 6.1% of total protein (Bremer and Dennis, 2008); the latter percentage results from multiplying 9.2% by the relative proportion of the masses of these other proteins to the mass of ribosomal proteins, 556/850 [calculated for cells with an MDT of 40 min from Table 4 in Bremer and Dennis (2008)]; hence, the protein component of the transcriptional-translational machinery (the rybosomes) is 15.3% of total protein, namely 32.7 μg (15.3% of 214 μg). The RNA component of the rybosomes is 43 μg (98% of 44 μg). This gives **75.7** μg for the rybosomes. The remaining proteins, the enzimes, make up **181.3** μg (214–32.7 μg). This leaves **117** μg for the miscellaneous material. Converting these amounts to unit masses in a cell of 4,000 units gives 810 rybosomes (4,000 × 75.7/374), 1,939 enzimes (4,000 × 181.3/374), and 1,251 miscellaneous.

Given that the ratio of stable RNA to total RNA synthesis is 52% at an MDT of 60 min, we choose the fast to slow rybosome ratio to be 1. Given the variation in the inactive pool of ribosomes, we allow the inactive rybosomes to make up 50% of the rybosomes (Dai et al., 2016). Given that DNA, lipids and peptidoglycan make up 3.1, 9.1, and 2.5% dry weight, respectively (Neidhardt and Umbarger, 1996), we choose 15% as the minimum value of miscellaneous material needed for the cell to be viable, that is, 600 units. We therefore used Maple to solve equations (11–15) by choosing the values of the kinetic constants to give a composition of the population that is close to the above experimental values. We then use the values of the kinetic constants in the Visual Basic program.

### Viability

Populations of real cells can sometimes contain cells that neither grow nor divide and are effectively dead. To allow for the production of such *non-viable* cells, we choose minimum and maximum requirements for the five classes of constituents. This is particularly important for the miscellaneous material, which includes the cell wall and DNA; if a cell has a constituent outside this range it is considered non-viable and unable to divide (though it can still grow). In choosing the values of the kinetic constants and the segregation coefficients, we avoid those values that lead to the production of non-viable cells.

### Implementation of the Model

A program was written in Visual Basic 6 to explore the model. This allows numerous characteristics of the cells and of subpopulations of cells to be determined along with the history of every cell (which is a record of the characteristics of the lineage of that cell). Each bacterial cell, i, which is inspected once per minute, is characterized by a set of 15 parameters, Cell(i,j), where, for a newborn cell, Cell(i,1) = the number of inactive rybosomes; Cell(i,2) = the number of fast rybosomes; Cell(i,3) = the number of slow rybosomes; Cell(i,4) = the number of enzimes; Cell(i,5) = the number of miscellaneous elements. Cell(i,6) = the time in minutes remaining to division, which is reduced by one every minute; Cell(i,7) = the MDT in minutes; Cell(i,8) = the nature of the parental strand (equaling 1 for the fast strand and 2 for the slow strand). Equations (16)-(20) are used to obtain: Cell(i,9) = a spare parameter used for the growth curves of a few cells that are chosen at the end of the program. Equations (16)–(20) are also used to obtain the composition that this cell will have when it is about to divide (i.e., at the time it becomes a mother cell): Cell(i,10) = the number of inactive rybosomes at the MDT; Cell(i,11) = the number of fast rybosomes at the MDT; Cell(i,12) = the number of slow rybosomes at the MDT; Cell(i,13) = the number of enzimes at the MDT; Cell(i,14) = the number of miscellaneous elements at the MDT; Cell(i,15) = the viability of the cell (equaling 1 for viable and 0 for non-viable).

The values of the composition parameters, Cell(i,1) to Cell(i,5), are obtained from the segregation of the material in the mother cell when Cell(i) is born (see above). This composition is then used to calculate the MDT in minutes of the cell [which is recorded in two places, Cell(i,6) and Cell(i,7)] and the future composition that this cell will have when it becomes a mother cell [Cell(i,10) to Cell(i,14)]. The nature of the parental strand that the new-born cell has inherited is also recorded at its birth as Cell(i,8). The second daughter cell, Cell(j) is created at the same time as Cell(i), Whilst Cell(i) has the same identity, i, as its parent, Cell(j) has a new identity. If a Cell(j) exists already the new values of the parameters for Cell(j) overwrite those of the old Cell(j) which is thereby lost from the turbidostat. The program then examines every cell once per minute. This examination entails reducing the time in Cell(i,6) by 1 min. Eventually Cell(i,6) equals zero, which means that a time equal to its MDT has elapsed and the cell is now ready to divide. The composition of Cell(i) as a mother cell, Cell(i,9) to Cell(i,14), is then used to generate two more daughter cells as above. Viability is determined when a cell is created and if the composition of the future mother is such that it falls outside the limits needed to make two viable daughters, the cell is labeled non-viable and is not allowed to divide (though it remains in the turbidostat until replaced).

### Coding the Inheritance Pattern

The history of the composition of every cell present in the system (i.e., its lineage) is stored. In the case of the strand inheritance pattern for a given lineage, there are two possibilities of parental strands (fast or slow) for each cell in the lineage. Hence, over five generations, there are 2^5^ possible combinations of a lineage of fast and slow strands. We assign each pattern a unique score based on a binary code of 0 for the fast strand and 1 for the slow strand over 5 generations. This entails multiplying the code for the strand inherited each generation by 2^g^ where *g* = 4 for the most recent generation (and *g* = 0 for the most distant generation). For example, a cell in the present population that inherits a fast strand from a mother in a cell line that over the previous generations had only inherited a slow strand would have a score of 15 (0. 2^4^ + 1. 2^3^ + 1.2^2^ +1.2^1^ + 1.2^0^); reciprocally, a cell that inherits a slow strand from a mother in a cell line that over the previous generations had only inherited a fast strand would have a score of 16 (1.2^4^ + 0. 2^3^ + 0.2^2^ +0.2^1^ + 0.2^0^).

## Results

### Strand Segregation of Hyperstructures Confers Diversity at a Medium Growth Rate

To calibrate our system, we choose values for the kinetic constants that give a bacterial composition roughly similar to that of *E. coli* growing in steady state with an MDT of 64 min in a defined medium at 37°C where no factor is limiting. A population of bacteria growing in our system without the strand segregation of hyperstructures has a CV of 1.7%, whereas populations with a proportion of a hyperstructure, P(seg), associating with either the fast or the slow strand have CVs of 14 and 16%, respectively, as shown in Table 1 [we set P(seg) = 0.7 because higher values can lead to the production of non-viable cells]. Surprisingly, this increase in growth rate diversity comes at very little cost insofar as the increase in the average MDTs is only 2–3 min. This means that a bacterial population using the strand segregation mechanism can have the advantage of increasing its growth rate diversity without the disadvantage of greatly decreasing its growth rate (and so not being able to outgrow a competing population).

**TABLE 1:** Segregation of hyperstructures causes diversity.

**Rate**	**Strand**	**k_IF_**	**P(seg)**	**MDT**	**SD**	**CV %**	**Fastest**	**Slowest**
Fast	Neither	0.07	0	50	0.7	1.4	48	53
	Both	0.07	0.7	51	2.5	4.9	44	56
	Fast	0.07	0.7	52	8.3	16	36	73
	Slow	0.07	0.7	51	6.3	12	40	65
Medium	Neither	0.05	0	64.1	1.1	1.65	61	67
	Both	0.05	0.7	64	2.9	4.5	59	69
	Fast	0.05	0.7	65	9	14	47	88
	Slow	0.05	0.7	66	11	16	47	93
Slow	Neither	0.008	0	313	17	5.5	268	380
	Both	0.008	0.7	332	84	25	170	639
	Fast	0.008	0.7	313	17	5.5	262	377
	Slow	0.008	0.7	340	97	29	151	707

*Three groups of growth rates were studied (Rate) with association of hyperstructures being with neither strand, both strands, the fast strand, or the slow strand. k_*IF*_, kinetic constant for the conversion of inactive into fast rybosomes; P(seg), proportion of hyperstructure associating with strand; MDT, mass doubling time of population; SD, standard deviation; CV, coefficient of variation; fastest, MDT of fastest cell; slowest, MDT of slowest cell.*

The presence of a low frequency of non-growing or slowly growing persisters in a growing bacterial population ensures that, after a stress has wiped out the rest of the population, a single persister can eventually grow again to recreate an identical population. It is therefore significant that the strand segregation mechanism can generate cells with MDTs as different as 47 and 93 min (Table 1). This means that for aspects of the phenotype in addition to the MDT such as resistance to different stresses, this mechanism could generate cells at both extremes.

### Increase in Diversity With Increased MDT

In the absence of the strand segregation mechanism, the distribution of cellular constituents to the daughter cells is random (within the defined limits for their maxima and minima). To investigate the effects of growth at different rates on diversity in the absence of this mechanism, we changed the kinetic constant, k_IF_, that determines the probability with which an inactive rybosome becomes a fast rybosome. Decreasing this constant from 0.07 to 0.05 to 0.008 results in corresponding MDTs of 50, 64.1, and 313 min and CVs of 1.4, 1.65, and 5.5% (“Neither” rows in Table 1). To explain this, we reasoned that (1) a new-born cell that has a composition differing from the steady state composition (which is fixed by the values of the kinetic constants) grows so as to converge on the steady state composition, (2) if it reaches this steady state composition on or before doubling its mass, there is little diversity in the MDTs (which depend on composition), (3) the probability of reaching the steady composition within an MDT depends essentially on the proportion of the new-born cell that are rybosomes, and (4) if the average number of rybosomes in the growth condition is small then a random distribution of rybosomes at division generates daughters with MDTs that differ more than if this average number is big [for example, if on 200,000 occasions six rybosomes are shared at random between two daughter cells some receive six and some none and their MDTs vary considerably whilst if 600 rybosomes are shared between two daughter cells all the daughters receive between 353 and 247 rybosomes and their MDTs are similar (Gangwe Nana et al., 2018)]. This was confirmed by the composition of the fastest and slowest growing cells in the three growth rate conditions (Table 2). In the fastest growth conditions (k_IF_ = 0.07), there are many rybosomes and cells at the extremes are close to the average composition (in other words, only a few division cycles are needed to converge on the steady state composition) whilst in the slowest growth conditions (k_IF_ = 0.008), there are few rybosomes and cells at the extremes are further from the average composition.

**TABLE 2:** Diversity of composition and MDTs increases at lower growth rates even without hyperstructures.

**Rate**	**k_IF_**	**Type**	**F**	**I**	**S**	**E**	**M**	**MDT**
Fast	0.07	Fastest	339	308	548	1899	910	48
	0.07	Average	311	284	528	1908	972	50.3
	0.07	Slowest	269	271	511	1934	1015	53
Medium	0.05	Fastest	190	231	466	1893	1220	61
	0.05	Average	177	215	421	1935	1254	64.1
	0.05	Slowest	147	199	397	1956	1300	68
Slow	0.008	Fastest	2	23	67	1075	2833	263
	0.008	Average	3	19	43	949	2988	313
	0.008	Slowest	1	11	27	815	3145	391

*Composition of the fastest, average, and slowest new-born cells in a population in the three growth rate conditions when cellular constituents were distributed at random to the daughter cells.*

### Strand Segregation Leads to Greater Increase in Diversity With Increased MDT

To investigate the effects of strand segregation at different growth rates, we again changed the kinetic constant, k_IF_, that determines the probability with which an inactive rybosome becomes a fast rybosome. Decreasing this constant from 0.05 to 0.008 results in an MDT of 313 min and a CV of 5.5% for a population growing slowly in the absence of the strand segregation mechanism (“Neither” row in Table 1). In the presence of this mechanism, the CV increases to as much as 29% for a population growing slowly with a hyperstructure associating with the slow strand (“Slow” row in Table 1); moreover, in this slow-growing population, the difference between the MDTs of the slowest and fastest cells is over four-fold as opposed to a mere one-third difference for a slow-growing population without the mechanism. Repeating this slow growth experiment with an entire hyperstructure segregating with the slow strand, that is with P(seg) = 1, results in a thirteen-fold difference in the MDTs of the slowest and fastest cells (not shown).

Increasing k_IF_ to 0.07 results in an MDT of 50 min and a CV of 1.4% for a population growing rapidly in the absence of the strand segregation mechanism (“Neither” in Table 1). In the presence of this mechanism acting on the fast strand with P(seg) = 0.7, the CV increases to 16%. However, in these fast growth conditions, the difference between the MDTs of the slowest and fastest cells is only two-fold as opposed to the 10% difference for the population without the mechanism (and as opposed to the four-fold difference for the slow growth condition, above).

**FIGURE 2:**
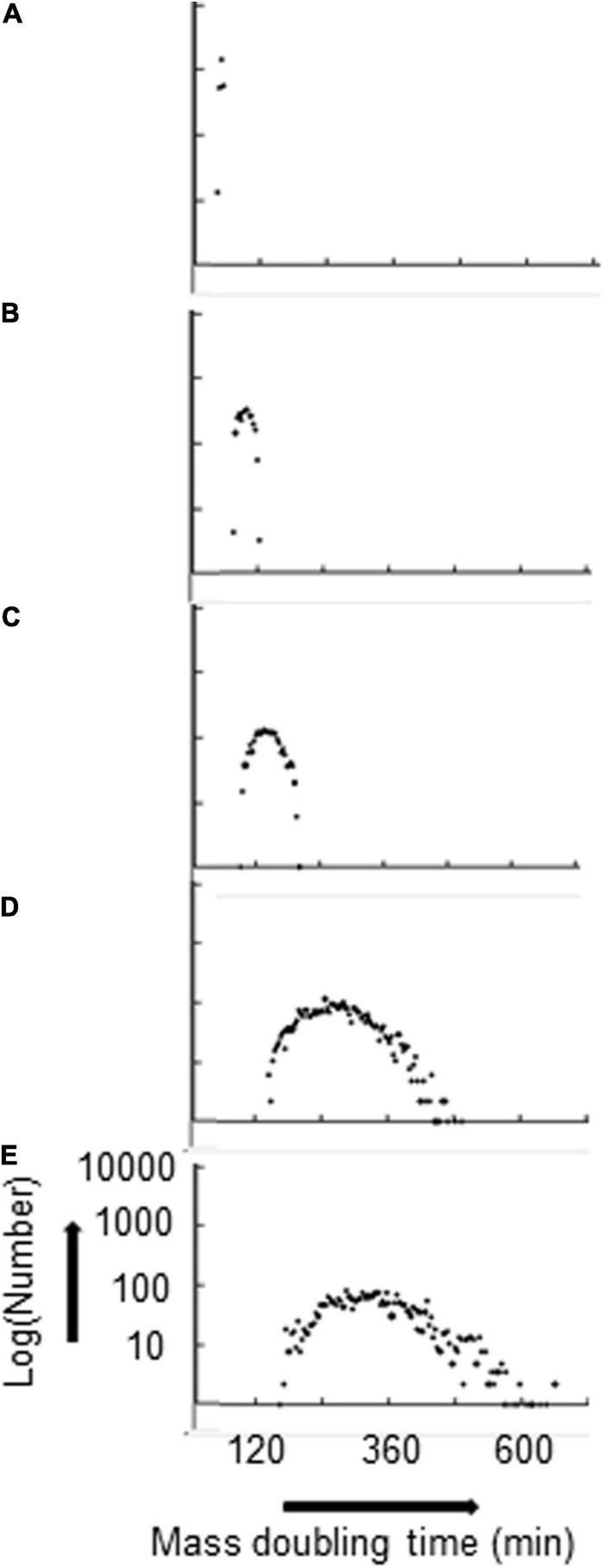
Distribution of MDTs broadens as the average MDT increases. Hyperstructures are segregated with a proportion P(seg) = 0.7 for both strands. k_IF_ and corresponding average MDT are: **(A)** 0.07, 51 min; **(B)** 0.03, 97 min; **(C)** 0.02, 140 min; **(D)** 0.01, 270 min; **(E)** 0.008, 333 min. The numbers are in bins of 4 mass doubling times.

With P(seg) = 0.7 for both the fast and the slow strands, the k_IF_ and the total number of rybosomes in the average cell decrease monotonically—for k_IF_ values of 0.07, 0.03, 0.02, 0.01, and 0.008, the total number of rybosomes in the average cell are 1,119, 452, 262, 92, and 65, respectively; this decrease in rybosome numbers is accompanied by a broadening distribution of the MDTs as shown in Figure 2. In other words, the strand segregation mechanism provides most diversity when cells are growing in poor conditions; this may be important because the transcriptional/translational capacity is limited in real cells growing in poor media where there is evidence that phenotypic diversity is most needed (Vohradsky and Ramsden, 2001).

### The Origins of Growth Rate Diversity in a Single Strand Hyperstructure

**FIGURE 3:**
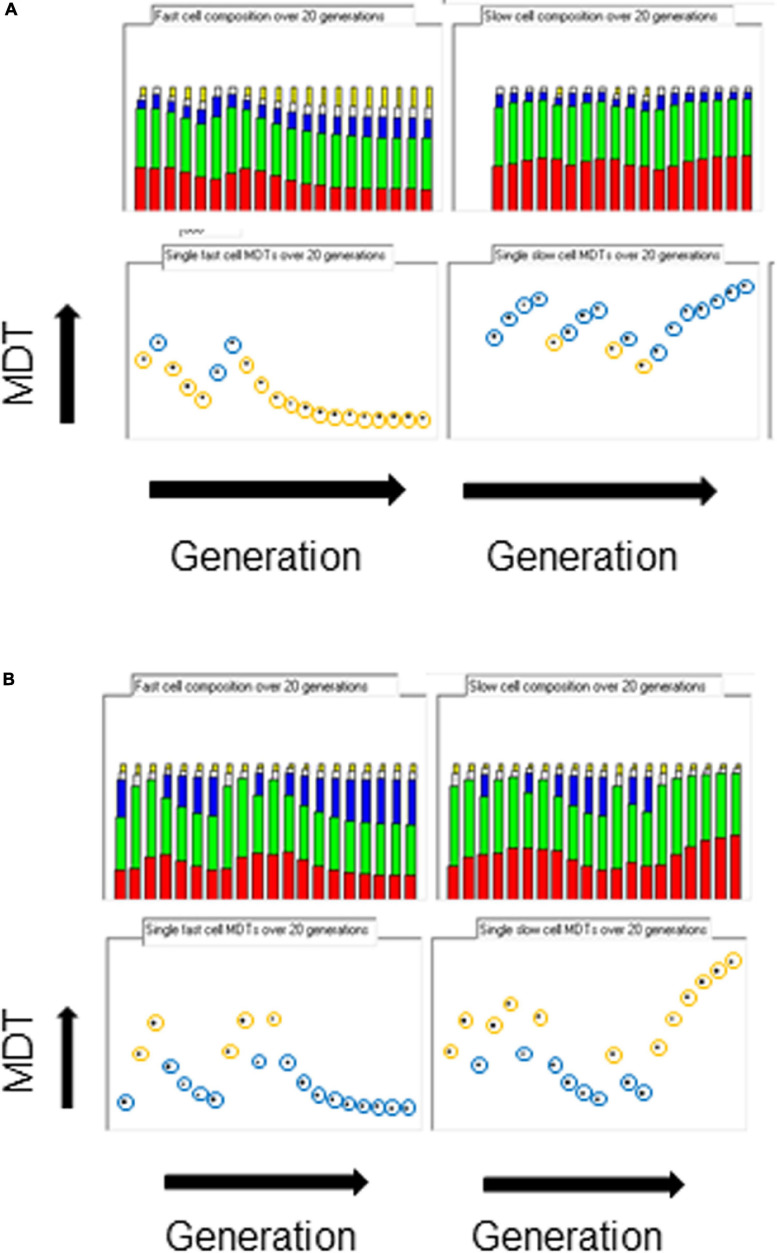
Origins of diversity. The composition of the fastest cell line (left columns) and the slowest cell line (right columns) over 20 generations in the medium growth rate condition (k_IF_ = 0.05). Top row, miscellaneous, enzimes, and slow, inactive and fast rybosomes (from bottom to top, red, green, blue, white, orange, respectively); bottom row, MDTs (orange circle = fast parental strand inherited; blue circle = slow parental strand inherited). **(A)** Only the fast hyperstructure is inherited. **(B)** Only the slow hyperstructure is inherited.

When the space of phenotypes is restricted to the space of growth rates, it could be argued that there are two attractors, one for flat-out growth in favorable conditions so as to distance competing cells and the other for slow or no growth in unfavorable conditions so as to survive. If the inheritance of the same parental strand and an associated hyperstructure over the generations could indeed be partly responsible for phenotypic diversity in real cells, examination of the fastest and slowest cells in the population should reveal such inheritance. Figure 3 shows the composition of the ancestors of these cells over twenty generations. In all growth conditions, when there is only a hyperstructure associated with the fast strand, the fastest growing cells have inherited the parental fast strand over several generations (the yellow circles in Figure 3A), which results in a corresponding increase in the rybosomal proportion of the mass; reciprocally, the slowest growing cells have inherited the corresponding slow strand over several generations (the blue circles in Figure 3A), which results in a corresponding increase in the non-rybosomal proportion of the mass.

The results are the opposite when the only hyperstructure in the cell is associated with the slow strand as shown in Figure 3B. Then, in all conditions, the fastest growing cells have inherited the slow strands and the slowest growing cells have inherited the fast strands; this is because in these conditions the cell that inherits the slow strand also inherits most of the active rybosomes. Fast- and slow-strand based segregation are alternative strategies for distributing rybosomes. When these strategies are combined (by having a hyperstructure associated with both strands), the result at the level of the population is that rybosomes are distributed more equally and diversity is less than when a hyperstructure is associated with just one strand; for example, this is the case at the medium growth rate where the CV is 4.5% for a population in which both strands associate with a hyperstructure but where the CV is 14% or 16% for populations in which only one strand associates with a hyperstructure (Table 1).

**FIGURE 4:**
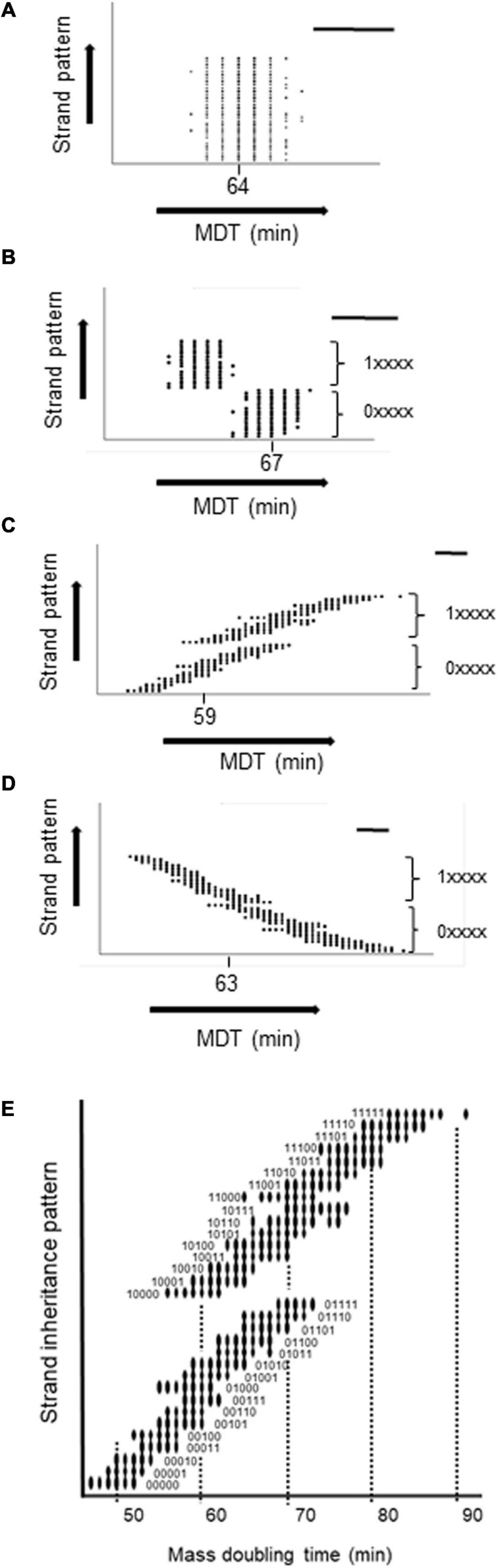
Relationship between the MDT of a cell and its strand inheritance in the medium growth conditions. The MDT of each cell in the population was plotted against its strand inheritance over the five previous generations with P(seg) = 0.7. Strand inheritance was coded as a 5-digit number in which each digit was either 0 (for the fast strand) or 1 (for the slow strand) giving a range from 0 to 31. The two major subpopulations resulting from the present cell inheriting a fast or slow strand from its mother are shown as 0xxxx and 1xxxxx. A circle indicates that there are one or more cells with the combination of an MDT and a particular inheritance pattern. **(A)** No inheritance. **(B)** Both fast and slow strands inherited. **(C)** Only the fast strand is inherited. **(D)** Only the slow strand is inherited. **(E)** Zoom of **(C)**. In each panel, the mode is shown and the bar = 5 min.

To confirm the generality of the relationship between the MDT and the pattern of strand inheritance of hyperstructures, we encoded this pattern as a binary score reflecting a cell’s lineage over five generations with 0 or 1 for inheritance of the fast strand or slow strand, respectively, and with the most recent generation having the greatest weight (so 0.2^4^ or 1.2^4^). Hence, a cell with a lineage of five generations of inheritance of only fast or only slow strands would have a score of 0 (0.2^4^ + 0. 2^3^ + 0.2^2^ +0.2^1^ + 0.2^0^) or of 31 (1.2^4^ + 1.2^3^ + 1.2^2^ + 1.2^1^ + 1.2^0^). We then plotted this score against the integer value of the MDT for each cell in the population as shown in Figure 4. It is apparent that (1) several subpopulations are created depending on the inheritance pattern, (2) the greatest effect on the MDT of a cell is its inheritance of a strand-associated hyperstructure from its mother (i.e., from the previous generation), (3) nonetheless, this effect can be outweighed by inheritance of a strand over the generations that preceded the mother as shown, for example, by the cells with short MDTs of 56, 59, and 61 min (corresponding to the fastest cells in patterns 10000, 10001, and 10010) compared to cells with the longer MDTs of 65, 65, and 69 (corresponding to the fastest cells in patterns 01101, 01110, and 01111), and (4) the most extreme MDTs are the cumulative result of cells inheriting a strand-associated hyperstructure over several generations as in the case of 47 and 91 min for patterns 00000 and 11111, respectively.

## Discussion

The ensemble of the above results supports the strand-dependent, hyperstructure-based hypothesis that coherent, growth rate diversity can be generated via the pattern of inheritance of strands of DNA and associated macromolecular assemblies as shown in Figure 1. These results are based on a model in which several simplifications are made. Firstly, there is only one type of hyperstructure for the strand-specific segregation of fast rybosomes and one type of hyperstructure for that of slow rybosomes: in reality, several hyperstructures serving related functions may segregate with a particular strand; hence, strand-based segregation could impose a coherent, essentially binomial, distribution of phenotypes. Secondly, the hyperstructures modeled here only act as hyperstructures in terms of the segregation of their constituents: in reality, hyperstructures have different half-lives, confer different activities on their constituents, interact with one another and follow different trajectories of birth, growth and decay (Norris et al., 2007); hence, the strand-based segregation of a subset of these hyperstructures offers rich possibilities for generating coherent diversity.

One cause of the growth rate diversity generated by the model is the small number of rybosomes at long MDTs. Low rybosome numbers mean that even a random distribution of rybosomes to daughter cells can generate considerable growth rate diversity (Table 2). The second cause is the strand-based segregation of a single hyperstructure containing many rybosomes, which results in a non-random distribution of rybosomes to daughter cells.

It might be thought that the fast strand, which is linked physically in the model to the autocatalytic transcriptional and translational machinery, would always confer a faster growth rate on the daughter cell inheriting it. This is not what we find with our model where the quantity of inherited rybosomes that determines the growth rate depends on whether these rybosomes are inherited via the fast strand or via the slow strand (Figure 3). In reality, ribosomal and related hyperstructures would accompany the parental strand encoding for example the ribosomal genes whilst other hyperstructures containing for example certain Nucleoid-Associated Proteins would accompany the other parental strand (Konto-Ghiorghi and Norris, 2020).

Natural selection is usually seen as acting on the genes that encode the macromolecules responsible for the kinetic constants so as to select, for example, a fast-growing mutant individual that out-competes other cells in rich media. In the strand-specific segregation hypothesis, natural selection can also act on the distribution of genes on the strands and on the association of macromolecules with one another and with the parental strands so as to select a population with a range of phenotypes simultaneously able to exploit or to resist environmental changes. In this strand-based segregation scenario, there is no specific selection for fast-growing mutants in rich medium since division of the fastest-growing cell generates both a fast-growing cell and a slower-growing cell—and the faster the fastest-growing cells grow, the faster they throw off these slower growers (Figure 1B). Instead, the selection is at the level of the factors responsible for generating a phenotypically diverse population (via the strand-based segregation of hyperstructures) whilst it is only the composition of the average cell that is determined by the values of the kinetic constants. This hypothesis is relevant to the origins of life given that at some early stage of evolution, cells appeared that replicated and segregated their DNA into daughter cells in what constitutes the cell cycle. Once this happened, cells possessed a powerful mechanism to generate a coherent phenotypic diversity that could operate in both prokaryotes and eukaryotes (Levin et al., 2016). Future evolution then built on and refined this fundamental mechanism by selecting a rich variety of regulatory systems.

The strand-dependent, hyperstructure-based hypothesis has implications for fundamental microbiology in that it proposes a mechanism whereby cells can adopt different, niche-specific strategies for generating coherent phenotypic diversity based on the quantity of the transcription-translation apparatus and on strand-specific inheritance. These implications extend to the hyperstructure approach to clinical microbiology where a “regrowth-delay” hyperstructure helps generate antibiotic-resistant bacteria (Yu et al., 2019).

The hypothesis could be tested using, for example, a *thyA* mutant of *E. coli* by pulse-labeling for a couple of minutes (1) the chromosomes with thymidine containing a stable isotope such as ^15^N and (2) proteins with amino acids containing a different stable isotope such as ^13^C. These cells could then be grown for several generations in a medium containing only ^14^N and^12^C. Analysis of these cells by *SIMS* should then reveal whether cells that have received the labeled parental strands have also received a disproportionate quantity of hyperstructures in the form of labeled proteins, as predicted by the hypothesis (Gangwe Nana et al., 2018). The nature of these hyperstructures could be further investigated by probing these cells with an appropriate aptamer or antibody to allow fluorescence microscopy before the *SIMS* step or, indeed, using an aptamer or antibody containing a third stable isotope to allow simultaneous quantification and localization of all isotopes by *SIMS*.

In conclusion, the strand-based segregation mechanism generates growth rate diversity at very little cost in terms of the overall growth rate of the population. Moreover, this mechanism provides most diversity at the slow growth rates where it is most needed. In allowing natural selection to provide a wide, coherent range of phenotypes early in evolution, the strand-based segregation of hyperstructures is, in a sense, the foundation of the phenotype.

## Data Availability Statement

The raw data supporting the conclusions of this article will be made available by the authors, without undue reservation.

## Author Contributions

VN wrote the Visual Basic program. CR wrote the Maple program. Both authors designed the model and wrote the manuscript.

## Conflict of Interest

The authors declare that the research was conducted in the absence of any commercial or financial relationships that could be construed as a potential conflict of interest.
